# The phenotype of the gut region is more stably retained than developmental stage in piglet intestinal organoids

**DOI:** 10.3389/fcell.2022.983031

**Published:** 2022-08-29

**Authors:** Eloïse Mussard, Corinne Lencina, Lise Gallo, Céline Barilly, Maryse Poli, Katia Feve, Mikael Albin, Laurent Cauquil, Christelle Knudsen, Caroline Achard, Guillaume Devailly, Laura Soler, Sylvie Combes, Martin Beaumont

**Affiliations:** ^1^ GenPhySE, Université de Toulouse, INRAE, ENVT, Castanet-Tolosan, France; ^2^ Lallemand Animal Nutrition, Blagnac Cedex, France; ^3^ Toxalim (Research Centre in Food Toxicology), Université de Toulouse, INRAE, ENVT, INP-Purpan, UPS, Toulouse, France

**Keywords:** enteroids, colonoids, gut segment, age, intestinal epithelium, microbiota, metabolome, epigenetics

## Abstract

Intestinal organoids are innovative *in vitro* tools to study the digestive epithelium. The objective of this study was to generate jejunum and colon organoids from suckling and weaned piglets in order to determine the extent to which organoids retain a location-specific and a developmental stage-specific phenotype. Organoids were studied at three time points by gene expression profiling for comparison with the transcriptomic patterns observed in crypts *in vivo*. In addition, the gut microbiota and the metabolome were analyzed to characterize the luminal environment of epithelial cells at the origin of organoids. The location-specific expression of 60 genes differentially expressed between jejunum and colon crypts from suckling piglets was partially retained (48%) in the derived organoids at all time point. The regional expression of these genes was independent of luminal signals since the major differences in microbiota and metabolome observed *in vivo* between the jejunum and the colon were not reproduced *in vitro*. In contrast, the regional expression of other genes was erased in organoids. Moreover, the developmental stage-specific expression of 30 genes differentially expressed between the jejunum crypts of suckling and weaned piglets was not stably retained in the derived organoids. Differentiation of organoids was necessary to observe the regional expression of certain genes while it was not sufficient to reproduce developmental stage-specific expression patterns. In conclusion, piglet intestinal organoids retained a location-specific phenotype while the characteristics of developmental stage were erased *in vitro*. Reproducing more closely the luminal environment might help to increase the physiological relevance of intestinal organoids.

## Introduction

The mammalian intestine is covered by a single layer of epithelial cells, which constitutes a barrier between the organism and the luminal environment (Peterson and Artis, 2014). Multipotent stem cells located at the base of crypt domains continuously renew the intestinal epithelium over 4–5 day periods (Gehart and Clevers, 2019). The epithelium plays diverse roles including digestion and absorption of nutrients, detection of pathogens and protection against harmful luminal components (Peterson and Artis, 2014). The multiple functions of the gut epithelium are supported by differentiated epithelial cells specialized for absorption (absorptive cells) or secretion of mucus (goblet cells), antimicrobial peptides (Paneth cells) and hormones (enteroendocrine cells) (Barker, 2014).

Recent advances in intestinal stem cell biology have enabled the *in vitro* culture of self-organizing three-dimensional epithelial structures called organoids (Sato et al., 2009). In contrast to cell lines, intestinal organoids are composed of primary non-transformed cells that can differentiate into each type of epithelial cell. This novel model is a powerful tool to study intestinal epithelium biology in various fields, including nutrition, host-microbe interactions, disease modeling and drug screening (In et al., 2016). Importantly, several studies have shown that intestinal organoids retain some of the characteristics of their tissue of origin, independently of the presence of mesenchyme or luminal signals (e.g. nutrients, microbiota and its metabolites). For instance, the epithelial phenotype observed in patients with digestive diseases are at least in part maintained in organoid culture, probably through an epigenetic imprinting of stem cells (Dotti et al., 2017; Howell et al., 2018; Freire et al., 2019; d’Aldebert et al., 2020).

A location-specific phenotype is also retained in human and mouse organoids derived from the small or large intestine, notably through the maintenance of a location-specific DNA methylation profile (Middendorp et al., 2014; Kraiczy et al., 2019; Kayisoglu et al., 2021). For instance, regional expression patterns of digestive enzymes and innate immune signaling components observed *in vivo* are maintained in organoids (Comelli et al., 2009; Kayisoglu et al., 2021). However, the *in vitro* maintenance of gut region characteristics is only partial and important differences are still observed at the transcriptome level between organoids and their tissue of origin (Middendorp et al., 2014; Kraiczy et al., 2019). It also remains unclear whether intestinal organoids retain the phenotype of their developmental stage of origin. Indeed, Senger et al. (2018) showed that the expression of developmentally regulated genes was stable in human fetal organoids. However, other studies demonstrated that fetal organoids undergo *in vitro* maturation, which was not observed in human and mouse organoids obtained after birth (Kraiczy et al., 2019; Navis et al., 2019). Interestingly, the expression of digestive enzymes in mouse fetal organoids matures over time *in vitro*, following a pattern characteristic of the changes observed *in vivo* at the suckling-to-weaning transition (Navis et al., 2019).

The methods used to culture human and mouse organoids were recently adapted to the pig intestine (Gonzalez et al., 2013; Khalil et al., 2016; Derricott et al., 2019; van der Hee et al., 2020; Beaumont et al., 2021a). The pig is an excellent model for studying the physiology of the human intestine due to greater similarities with this species compared to rodents regarding nutrition, gut structure, functionality and microbiota (Heinritz et al., 2013). Moreover, well characterized *in vitro* models of the pig intestinal epithelium are required for veterinary research (Beaumont et al., 2021a). Pig intestinal organoids retain to some extent a region-specific phenotype (van der Hee et al., 2020; Barnett et al., 2021) but these studies did not consider inter-individual variability since organoids were derived from a limited number of piglets (n = 2–3). Mohammad et al. (2020) showed that jejunum organoids derived from suckling and weaned piglets failed to retain the age-specific expression of digestive enzymes observed *in vivo*. However, it is not known whether pig organoids retain the major alterations of the epithelial barrier function observed at the suckling-to-weaning transition in piglets (Moeser et al., 2017). Moreover, a detailed characterization of the luminal environment (metabolome, microbiota) from which pig organoids originate is lacking, while it could be helpful to generate hypothesis regarding external factors required in the culture medium to maintain the original phenotype *in vitro*.

In this study, we generated jejunum and colon organoids from twelve suckling and twelve weaned piglets. Indeed, jejunum and colon have major functional and microbiota differences and the suckling-to-weaning transition is a key step of intestinal maturation. Our objective was to compare the gene expression profiles *in vivo* in epithelial crypts and *in vitro* in organoids at three time points to determine the extent to which organoids retained a regional and developmental stage-specific phenotype. We also analyzed the luminal metabolome and microbiota composition in order to characterize the environment of the intestinal stem cells at the origin of organoids.

## Material and methods

### Animals and sample collection

All animal experimentation procedures were approved by the local ethics committee (N°TOXCOM/0136/PP) in accordance with the European directive on the protection of animals used for scientific purposes (2010/63/EU). Piglets were obtained from a local farm (Gaec de Calvignac, Saint Vincent d'Autejac, France). From birth to day 21, piglets were exclusively suckling. Between day 21 and day 28, piglets were suckling and had access to a commercial solid starter diet for piglets (Belecla, CCPA). At day 28, piglets were weaned and moved in an experimental animal facility (Toxalim, INRAE, Toulouse, France) where they had access to the same commercial solid feed as prior to weaning. Samples were collected at day 21 from twelve suckling piglets (n = 6 castrated males, n = 6 females, mean body weight: 7.05 kg, s. e.m: ± 0.30 kg) and at day 35 from twelve weaned piglets (n = 6 castrated males, n = 6 females, mean body weight: 9.07 kg, s. e.m: ± 0.33 kg) (Supplementary Figure S1). The experiments were divided into three batches of suckling piglets and three batches of weaned piglets. Piglets were slaughtered by electronarcosis followed by exsanguination. Jejunum (middle part of the small intestine between the stomach and the caecum) and colon (central flexure) tissues were collected and stored in ice-cold PBS (Fisher Scientific, cat#10010–056) supplemented with 1% penicillin/streptomycin (Sigma-Aldrich, cat#P4333) before processing as described below. Jejunum and colon contents adjacent to the sampling site of tissues were collected and stored at −80°C until analysis.

### Isolation of intestinal crypts

Jejunum and colon tissues (∼2–3 cm in length) were opened longitudinally and washed with cold PBS. Tissues were transferred to a Petri dish filled with cold PBS and gently scraped with a microscope slide to remove mucus and jejunum villi before transfer to a dissociation solution [PBS containing 9 mM EDTA (Fisher Scientific, cat# AM9260G), 3 mM 1,4-Dithiothreitol (DTT) (Sigma-Aldrich, cat# 10197777001), 10 μM ROCK inhibitor Y27632 (ATCC, cat#ACS-3030] and 1% penicillin/streptomycin). After 30 min of incubation at room temperature on a rotating shaker, tissues were transferred in a Petri dish filled with cold PBS. Crypts were isolated from the tissue by firm scraping with a microscope slide and filtered with a 100 μm cell strainer. After centrifugation (500 g, 5 min, 4°C) the pellet was re-suspended in cold Dulbecco’s Modified Eagle Medium (DMEM, Fisher Scientific, cat#31966047) supplemented with 10 µM Y27632 and crypts were counted with an hemocytometer. An aliquot was centrifuged (500 g, 5 min, 4°C) and the crypt pellet was lyzed in TRI Reagent (Zymo Research, cat# R2050) and stored at −80°C until RNA purification.

### Intestinal organoid culture

Crypts were seeded at a density of 150 crypts per 25 µL of Matrigel (Corning, Cat #354234) in pre-warmed 48-well plates. After polymerization (20 min, 37°C, 5% CO_2_), 250 µL of IntestiCult Organoid Growth Medium (Human) (STEMCELL Technologies, cat#06010) supplemented with 1% penicillin/streptomycin and 10 µM Y27632 was added. The medium was replaced every 2–3 days, without Y27632. Jejunum organoids were obtained from all suckling and weaned piglets (n = 12 per group). Colon organoids were obtained from all suckling piglets (n = 12) but from only five weaned piglets, due to a high rate of contamination probably derived from the gut microbiota. Thus, we did not include colon organoids from weaned piglets in our analyses. Previous studies reported the successful growth of colon organoids from weaned pigs (Barnett et al., 2021), indicating that our protocol was suboptimal for the colon of weaned piglets (washing procedure or antibiotics used). Organoids were subcultured at day 5 (passage 1, P1) and at day 9 (passage 2, P2) after seeding. For passaging, organoids were washed with warm PBS, broken by pipetting in EDTA-trypsin 0.25% w/v (Fisher Scientifc, cat#11560626) before incubation (5 min, 37°C, 5% CO_2_) and centrifugation (300 g, 5 min, room temperature). The pellet was re-seeded in Matrigel with a dilution ratio between 1:5 and 1:8 (according to the density of the organoids) and cultured as described before without Y27632. At post-seeding day 5 (P0), 9 (P1) and 13 (P2), organoids were counted according to their spherical or non-spherical morphology by bright field optical microscopy (Eclipse Ts2, Nikon Instruments) and with the ImageJ Software. At post-seeding day 5 (P0), 9 (P1) and 13 (P2), organoids (pool of six wells) were washed with PBS and lyzed in TRI Reagent before storage at −80°C until RNA purification. Organoids were cryoconserved at P1 by pooling 4-6 Matrigel domes in freezing medium (80% DMEM, 10% DMSO, 10% fetal bovine serum (FBS, Fisher Scientific cat#10270106), 10 µM Y27632) before transfer in a cryotube and freezing in CoolCell LX (Corning, cat# 432138) at −80°C until transfer in liquid nitrogen for long term storage.

### Differentiation of organoids

Organoids frozen at P1 were thawed and cultured for 1 week before passaging as described above. After 7 days of culture in IntestiCult Organoid Growth Medium (Human), the medium was replaced for 2 days by a differentiation medium lacking niche factors (DMEM supplemented with 10% FBS and 1% penicillin/streptomycin). At day 9, organoids (pool of six wells) were washed with PBS and lyzed in TRI Reagent before storage at −80°C until RNA purification.

### Gene expression

Gene expression was analyzed in epithelial crypts and in organoids at P0, P1, P2 and in the differentiated organoids. Total RNA from crypts and organoids was purified with the Direct-zol RNA MiniPrep Plus kit (Zymo Research, cat#R2072) according to the manufacturers instructions. A DNAse I digestion step was included to remove genomic DNA. After elution in RNase-free water, RNA concentration was determined using a Nanodrop and cDNA were prepared from 500 ng RNA with the GoScript Reverse Transcription Mix, Random primer (Promega, cat# A2801) following the manufacturers instructions. cDNA were diluted 1:2 (v/v) in DNAse/RNAse free water and stored at −20°C. Gene expression was analyzed by real-time qPCR using QuantStudio six Flex Real-Time PCR System (Thermofisher) and Biomark microfluidic system using 96.96 Dynamic Arrays IFC for gene expression (Fluidigm) according to the manufacturers recommendations. The sequences of the primers used are presented in Supplementary Table S1. Data were normalized to the stably expressed genes *RPL32* (crypts and organoids at P0, P1 and P2) or *GAPDH* (differentiated organoids) and analyzed with the 2^−ΔCt^ method.

### Chromatin immunoprecipitation (ChIP)-qPCR

Jejunum and colon organoids from five suckling piglets cryopreserved at passage one were thawed and cultured for 1 week before passaging as described above. ChIP was performed 7 days post-seeding. Organoids in matrigel were washed in PBS and dissociated into single cells by pipetting after incubation in EDTA-trypsin 0.25% w/v for 15 min at 37°C. ChIP targeting H3K4me3 was performed with 500 000 cells from each organoid line with the True MicroChIP-seq Kit (Diagenode, Cat#C01010132), following the manufacturers instructions. Briefly, protein-DNA were cross-linked for 8 min with 1% paraformaldehyde before quenching with glycine, cell lysis and fragmentation of the cross-linked chromatin using a Bioruptor Pico (Diagenode) with six cycles (30 s ON/OFF). An input of each sample was saved before immunoprecipitation. H3K4me3-DNA complexes were immunoprecipitated with an antibody targeting H3K4me3 (0.5 µg per reaction, provided in the kit) and protein-A magnetic beads. Cross-links were reversed and DNA was purified using DiaPure columns and eluted in 35 µL DNA elution buffer. qPCR was performed on input (diluted 1:10) and immunoprecipitated DNA with primers targeting the transcription start site (TSS) of the housekeeping gene HPRT (F: 5′-GGC​ATG​CGT​GGA​CTG​GTA​TT-3′, R: 5′-TGA​GGA​TGC​AAC​GAG​GCA​AT-3′), TLR4 (F: 5′-TGC​TTT​CTC​CGG​GTC​ACT​TC-3′, R: 5′-AAG​GGT​CCC​AGC​TCT​CAG​AT-3′) and LYZ (F: 5′-CCA​GAG​CTC​CGA​GAC​AAC​AG-3′, R: 5′-CGG​CGG​TTT​CTT​TTG​TGT​GT-3′) and intronic regions of TLR4 (F: 5′-CCT​CTG​ATG​GAT​GAG​CTG​CC-3′, R: 5′-AGC​AAT​GAA​GAG​GCC​CAC​AA-3′) and LYZ (F: 5′-CCA​TAC​GGA​GGC​CAG​AAG​AC-3′, R: 5′-GGA​GTT​GAA​GCG​ACA​CTC​CT-3′), used as negative controls. qPCR was performed using a QuantStudio six real-time PCR detection system (Thermo Fisher Scientific). The results were expressed as 2^ΔΔCt^ with ΔΔCt=(Ct_Input_-Ct_H3K4me4_)_Target gene_—(Ct_Input_-Ct_H3K4me4_)_Reference gene_. The gene expression of *TLR4* and *LYZ* were analyzed in the organoids used for ChIP as described above.

### Histological analysis

Transverse sections of jejunum and colon tissues with luminal content were fixed in Carnoy’s solution (60% ethanol, 30% chloroform, 10% glacial acetic acid) for 3 h before transfer in 70% ethanol, embedding in paraffin and staining with Alcian blue and Periodic Acid Schiff at the histology platform Anexplo (Genotoul, Toulouse, France). Slides were digitalized and villus height and crypt depth were measured with the CaseViewer 2.3 software (3DHISTECH). The measurements were repeated twice by two independent investigators blinded to the groups.

### 16S rRNA gene sequencing and sequence analysis

DNA was extracted from jejunum and colon contents using the Quick-DNA™ Fecal/Soil Microbe Miniprep Kit (Zymo Research, cat# 6010) and the 16S rRNA gene V3−V4 region was amplified by PCR and sequenced by MiSeq Illumina Sequencing as previously described (Verschuren et al., 2018). Sequencing reads were deposited in the National Center for Biotechnology Information Sequence Read Archive (SRA accession: PRJNA800253). 16S rRNA gene amplicon sequences were analyzed using the FROGS pipeline (version 3.2) according to standard operating procedures (Escudié et al., 2018). Amplicons were filtered according to their size (350–500 nucleotides) and clustered into OTUs using Swarm (aggregation distance: step 1 with d = 1 and step 2 with d = 3). After chimera removal, OTUs were kept when representing more than 0.005% of the total number of sequences and were shared by at least four individuals (Bokulich et al., 2013). OTUs were classified using the reference database silva138.1 16S with a pintail quality of 100 (Quast et al., 2013). The mean number of reads per sample was 33,916 (min: 23,731–max: 46,485).

### Nuclear magnetic resonance metabolomics

The metabolome was analyzed in jejunum and colon content (50 mg) by using nuclear magnetic resonance (NMR) at the MetaboHUB-MetaToul-AXIOM metabolomics platform (Toulouse, France) as described previously (Beaumont et al., 2021b). Annotated representative spectra in each group are presented in Supplementary Figure S2. For each of the 34 identified metabolites, buckets non-overlapping with other metabolites were selected for quantification (Supplementary Table S2).

### Statistical analyses

All statistical analyses were performed using the R software (version 4.1.1). The microbiota composition analysis was performed using the Phyloseq package (version 1.36.0) (McMurdie and Holmes, 2013). For *a* and *ß* diversity analyses, the samples were rarefied to even sequencing depth (23,731 reads per sample). Observed OTUs and Shannon *a*-diversity indices were calculated. The *ß*-diversity was analyzed using the Bray-Curtis distance and plotted by non-Metric Dimensional Scaling (nMDS). OTUs unrarefied counts were agglomerated at phylum or family level and relative abundances were calculated at each taxonomic level. Metabolome and gene expression data were log-10 transformed while the bacterial relative abundance data were transformed to the power of 0.25. Gene expression data were analyzed separately in crypts and in organoids at each passage (P0, P1, P2). For samples in which the expression of some genes was undetectable, half of the lowest expression value measured for these genes was imputed. We focused our analyses on two comparisons: 1) jejunum versus colon in suckling piglets (gut region effect) and 2) jejunum of suckling versus weaned piglets (developmental stage effect). All statistical analyses were performed with linear mixed models (R packages lme4, car and emmeans) with the gut region (jejunum or colon) or developmental stage (suckling or weaned) as fixed effect, while piglets and sampling batch were random effects. No effect of sex was observed in our data, and was thus not considered in statistical analyses. *p*-values were adjusted for multiple-testing with the Benjamini and Hochberg method. For ChIP-qPCR experiments, paired t-tests were used to compared jejunum and colon for each genomic region. Differences were considered significant when *p*-value was ≤0.05. PCA were performed with the mixOmics package (version 6.16.3) (Rohart et al., 2017). The heatmaps were created using the pheatmap package (version 1.0.12) with complete method algorithm and Euclidean distances for clustering.

## Results

### A location-specific phenotype is retained in jejunum and colon organoids from suckling piglets

**FIGURE 1:**
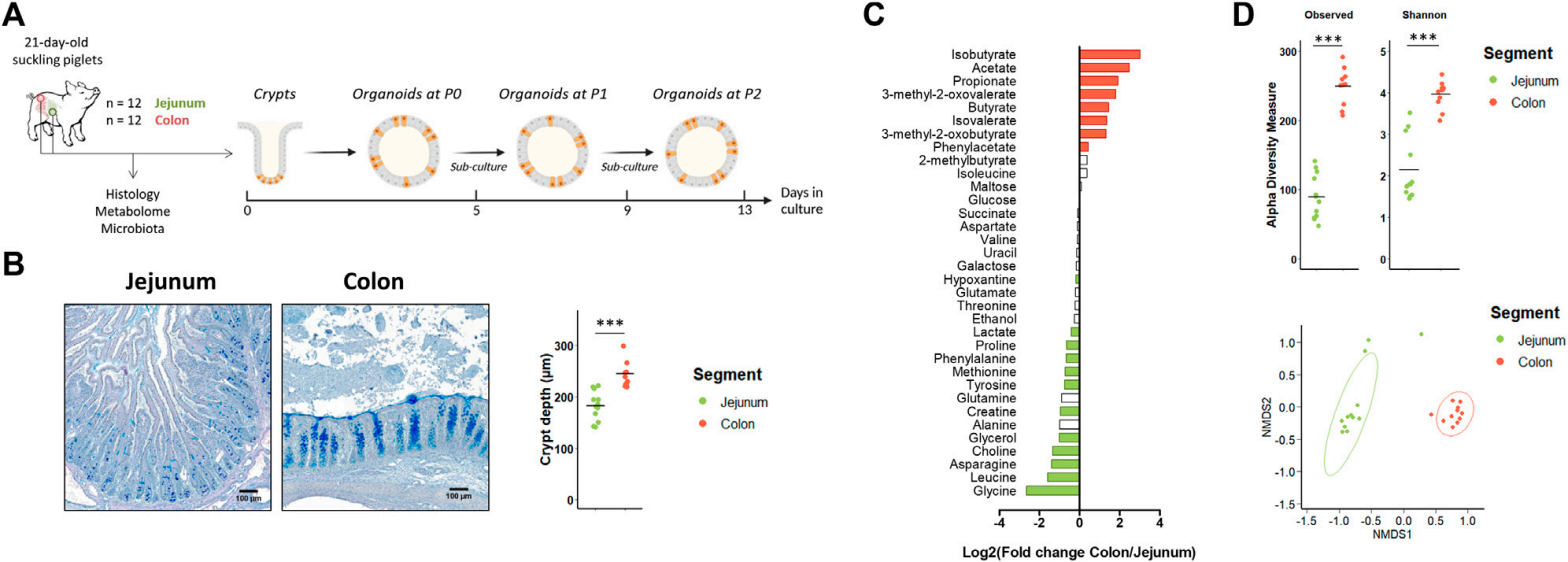
*In vivo* characteristics of jejunum and colon in 21-day-old suckling piglets. **(A)**. Experimental design for the culture of jejunum and colon organoids from 21-day-old suckling piglets (*n* = 12 per group). **(B)**. Histological observations and crypt depth of jejunum and colon sections stained with Alcian blue and Periodic Acid Schiff. Scale bars represent 100 µm. **(C)**. Mean Log2 fold change of the relative abundance of metabolites in colon (*n* = 11) versus jejunum (*n* = 12). Significant differences (*p* < 0.05) are indicated in red (metabolites more abundant in colon than in jejunum) or in green (metabolites more abundant in jejunum than in colon). **(D)**. Observed OTUs and Shannon *a*-diversity index and Non-Metric Dimensional Scaling (nMDS) two-dimensional representation of the microbiota *ß*-diversity using Bray-Curtis distance calculation (stress = 0.06) in the jejunum (*n* = 12) and colon (*n* = 11) contents. ***: *p* < 0.001.

Our first objective was to determine if jejunum and colon organoids from suckling piglets retained a location-specific phenotype (Figure 1A). *In vivo,* we observed, as expected, major differences in morphology, metabolome and microbiota between the jejunum and the colon of suckling piglets. Villi were present only in the jejunum and crypts were deeper in the colon than in the jejunum (Figure 1B). The luminal relative concentration of several amino acids, choline, glycerol, creatine and lactate was higher in the jejunum while the concentration of gut microbiota-derived metabolites was higher in the colon (Figure 1C and Supplementary Table S3). The gut microbiota *a*-diversity indices were higher in the colon than in the jejunum and a distinct microbiota structure was observed in each segment (Figure 1D). Lactobacillaceae (73%) was the dominant family in the jejunum while Lachnospiraceae (15%), Lactobacillaceae (13%), Oscillospiraceae (13%) and Prevotellaceae (12%) were the dominant families in the colon (Supplementary Table S4).

**FIGURE 2:**
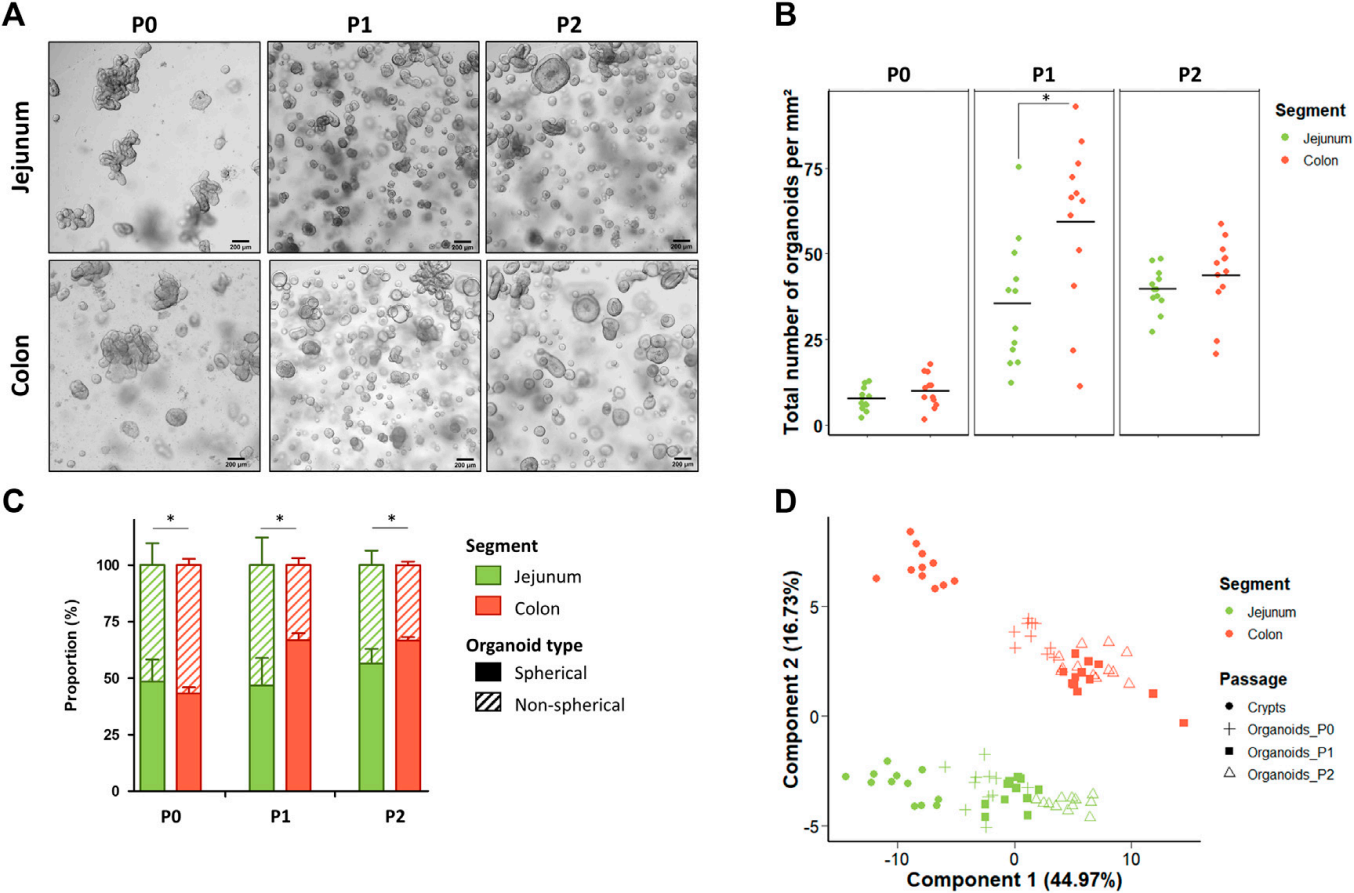
Characteristics of jejunum and colon organoids derived from 21-day-old suckling piglets. **(A)**. Representative images showing jejunum and colon organoids from 21-day-old suckling piglets at each passage. The scale bar represents 200 µm. **(B)**. Total number of jejunum and colon organoids per mm^2^ at each passage (*n* = 12 per group). **(C)**. Proportion of jejunum and colon organoids according to their spherical or non-spherical morphology at each passage (*n* = 12 per group). **(D)**. Principal component analysis (PCA) plot of the expression of 89 genes in crypts (*n* = 12 jejunum, *n* = 11 colon) and organoids at primary culture (*n* = 11 jejunum, *n* = 10 colon), passage 1 (*n* = 11 jejunum, *n* = 12 colon) and passage 2 (*n* = 12 jejunum, *n* = 12 colon). *: *p* < 0.05.

Jejunum and colon organoids were obtained from all suckling piglets (n = 12) (Figure 2A). The density of organoids derived from the jejunum and the colon was similar in the primary culture (P0) (Figures 2A,B). At passage 1 (P1), the density of organoids increased and was higher in organoids derived from the colon than from the jejunum while no difference was observed between segments at passage 2 (P2). Spherical and non-spherical organoids were obtained from the jejunum and colon at each passage. At P0, organoids were more spherical in the jejunum than in the colon, while at P1 and P2 organoids were more spherical in the colon (Figure 2C).

We analyzed the gene expression of 89 genes in jejunum and colon epithelial crypts from suckling piglets and in the derived organoids at P0, P1 and P2. PCA revealed a distinct gene expression profile between the jejunum and the colon and between crypts and organoids (Figure 2D). Interestingly, the gene expression profile observed in crypts appeared more similar to the expression patterns observed in organoids at P0 than at later passages.

**FIGURE 3:**
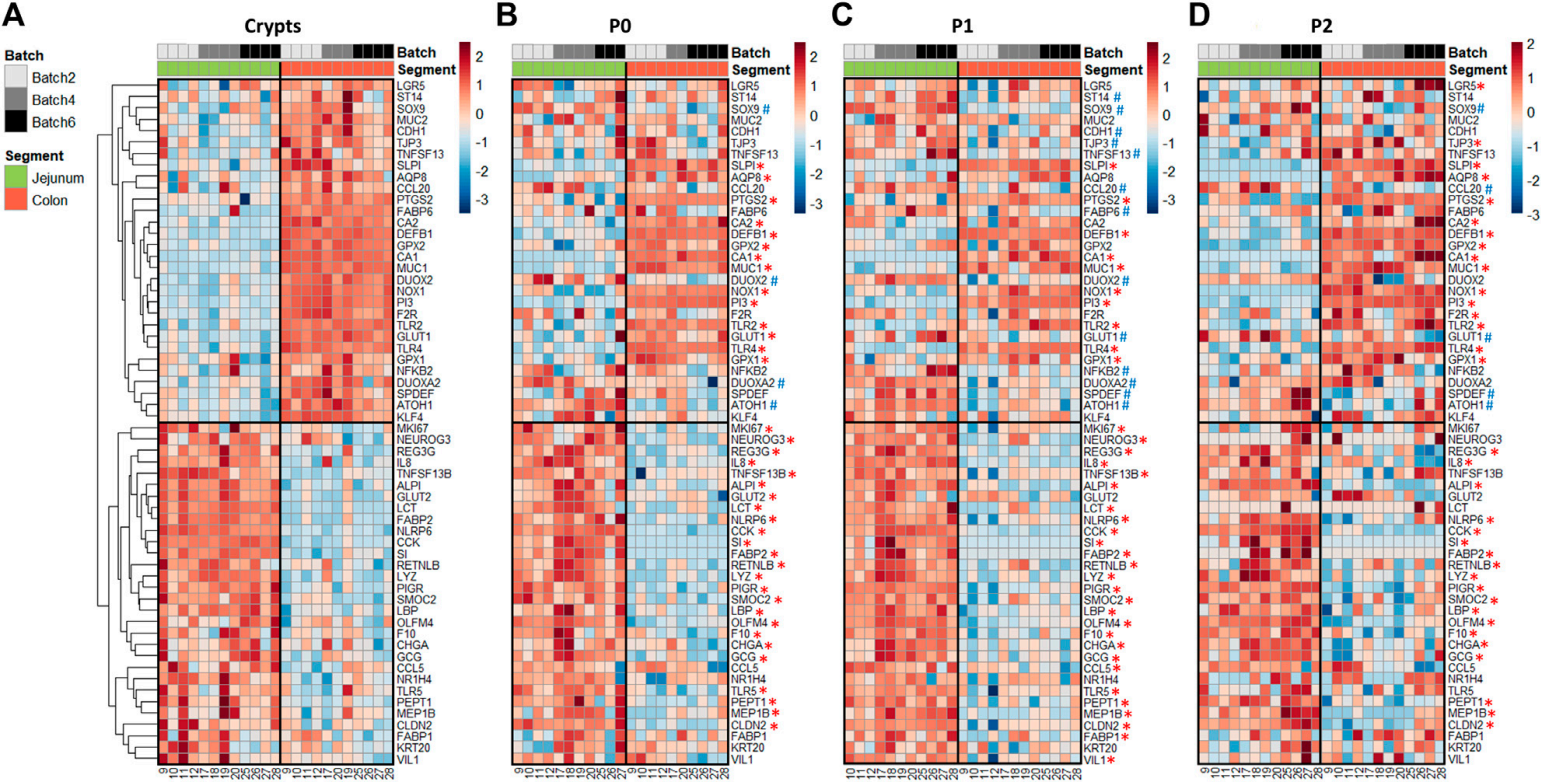
Gene expression profiles in jejunum and colon crypts and organoids from 21-day-old suckling piglets. Heatmap representing the relative expression of genes (rows) in each 21-day-old suckling piglet (columns) in jejunum and colon crypts (*n* = 12 jejunum, *n* = 11 colon) **(A)**, organoids at primary culture (n = 11 jejunum, *n* = 10 colon) **(B)**, passage 1 (*n* = 11 jejunum, *n* = 12 colon) **(C)** and passage 2 (*n* = 12 jejunum, *n* = 12 colon) **(D)**. The numbers at the bottom are piglet identifiers. The colors represent the Z-scores (row-scaled relative expression) from low (blue) to high values (red). Genes (rows) were clustered in crypts and presented in the same order in organoids. *: significant difference in organoids with the same direction as observed in crypts. #: significant difference in organoids in the opposite direction of the observed in crypts.

In crypts, 60 out of the 89 genes analyzed (67%) were differentially expressed between the jejunum and the colon of suckling piglets (Figure 3A and Supplementary Table S5). Genes more expressed in the jejunum crypts (30 differentially expressed genes, DEG) coded for markers of differentiated enterocytes (*ALPI, VIL1, KRT20*), digestive enzymes (*SI, LCT*), enterohomones (*CCK, GCG, CHGA, NEUROG3*), transporters and sensors of nutrients (*FABP1, FABP2, PEPT1, NR1H4, GLUT2*), antimicrobial peptides (*LYZ, REG3G, RETNLB*), proteins involved in innate immunity (*TLR5, LBP, IL8, CCL5, NLRP6*), proteins involved in immunoglobulin secretion (*PIGR, TNSF13B*), proteins involved in proteolytic activity (*F10, MEP1B*), a tight junction protein (*CLDN2*) and stem cell and proliferation markers (*SMOC2, MKI67, OLFM4*). Genes more expressed in the colon crypts (30 DEG) coded for markers of colonocytes (*CA1, CA2, AQP8*), an antimicrobial peptide (*DEFB1*), proteins involved in innate immunity (*TLR2, TLR4, NFKB2, CCL20*), proteins involved in mucus production (*MUC1, MUC2, SPDEF, KLF4, ATOH1*), redox enzymes (*PTGS2, NOX1, GPX1, GPX2, DUOX2, DUOXA2*), proteins involved in proteolytic activity (*PI3, SLPI, F2R, ST14*), cell junction proteins (*TJP3, CDH1*), a cytokine involved in immunoglobulin secretion (*TNFS13*), nutrient transporters (*GLUT1, FABP6*) and stem cell markers (*SOX9, LGR5*).

In organoids, 48% of the DEG identified *in vivo* were also differentially expressed at each time point (P0, P1 and P2) between jejunum and colon organoids from suckling piglets (Figures 3B–D and Supplementary Table S5). All of these DEG showed the same direction *in vivo* and *in vitro,* when comparing the jejunum and colon. DEG identified both in crypts and in organoids covered a broad range of epithelial functions: markers of enterocytes/colonocytes (*CA1, ALPI*), digestive enzymes and transporters of nutrients (*SI, FABP2, PEPT1*), enterohormones (*CCK, GCG, CHGA*), antimicrobial peptides (*LYZ, REG3G, DEFB1, RETNLB*), a mucin (*MUC1*), proteins involved in innate immunity (*TLR2, TLR4, LBP, IL8, NLRP6*), redox enzymes (*NOX1, PTGS2, GPX1*), proteins involved in proteolytic activity (*PI3, SLPI, F10, MEP1B*), an immunoglobulin transporter (*PIGR*), a cell junction protein (*CLDN2*) and stem cell markers (*SMOC2, OLMF4*).

In contrast, 52% of the DEG identified *in vivo* when comparing jejunum and colon epithelial crypts from suckling piglets were either 1) maintained between jejunum and colon organoids at some but not all passages (27% of DEG), 2) maintained between jejunum and colon organoids at all passages but with an opposite direction when compared to crypts (3% of DEG), 3) not maintained between jejunum and colon organoids (22% of DEG). Genes whose location-specific expression profile was partially or totally erased *in vitro* were involved in several epithelial functions including digestion, nutrient transport and sensing (*LCT*, *GLUT1, GLUT2, FABP1, FABP6, NR1H4*), enterohormone secretion (*NEUROG3*), innate immunity (*NFKB2, TLR5, CCL5, CCL20*), immunoglobulin secretion (*TNFSF13, TNFSF13B*), mucus secretion (*MUC2, SPDEF, KLF4, ATOH1*), redox homeostasis (*DUOX2, DUOXA2, GPX2*), proteolytic activity (*F2R, ST14*), cell junctions (*TJP3, CDH1*), epithelium renewal (*MKI67, SOX9, LGR5*) and markers of enterocytes/colonocytes (*VIL1*, *AQP8, CA2*) or differentiation (*KRT20*).

We hypothesized that the loss of some location-specific gene expression patterns might be due to a low differentiation level in organoids when cultured in IntestiCult medium which is known to promote proliferation (Zietek et al., 2020). Thus, we thawed a subset of jejunum and colon organoids from suckling piglets (n = 4/group) and cultured them for 2 days in a differentiation medium lacking niche factors. We focused our analysis on genes which *in vivo* regional expression was not retained in organoids cultured in IntestiCult at P2 (Figure 3D). The expression of *LCT*, *GLUT2*, *VIL1* and *CCL5* was higher in differentiated organoids derived from jejunum than from colon, which corresponds to the regional expression pattern observed *in vivo* (Supplementary Figure 3A–B and Figure 3A). In contrast, the location-specific expression of *NEUROG3*, *DUOX2*, *MUC2* and *KLF4* was not observed in differentiated organoids. These results indicate that promoting differentiation in organoids might help to reproduce the location-specific gene expression pattern of some but not all genes.

As a next step, we explored the epigenetic regulations underlying the regional expression of *TLR4* and *LYZ* in a subset of jejunum and colon organoids of suckling piglets (n = 5 per group). We performed ChIP-qPCR to quantify the trimethylation of histone H3 lysine 4 (H3K4me3), a mark associated with gene activation (Supplementary Figure 4). As expected, H3K4me3 was higher in the promoter (TSS) of *TLR4* and *LYZ* when compared to intronic regions. The higher mRNA level of *TLR4* in colon organoids when compared to jejunum organoids was associated with higher H3K4me3 in *TLR4* TSS in four out of five piglets, but this result was not statistically significant. The higher mRNA level of *LYZ* in jejunum organoids when compared to colon organoids was not correlated with H3K4me3 in *LYZ* TSS. Thus, our result suggest that H3K4me3 is not the main driver of the regional expression of *TLR4* and *LYZ* in organoids.

Altogether, our results show that jejunum and colon organoids from suckling piglets retained a partial location-specific gene expression signature. The maintenance of the gene expression pattern of the gut region in organoids appeared to be gene-specific rather than epithelial function-specific and is influenced by the culture conditions.

### Developmental stage-specific phenotype is not stably maintained in jejunum organoids derived from suckling and weaned piglets

**FIGURE 4:**
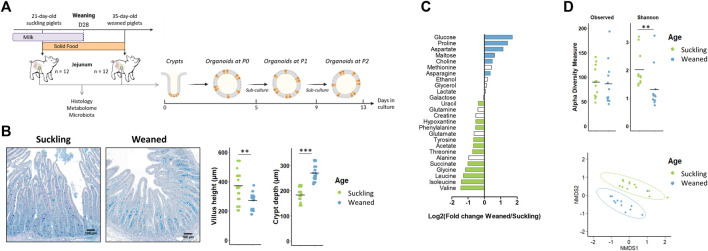
*In vivo* characteristics of jejunum from 21-day-old suckling and 35-day-old weaned piglets. (A). Experimental design and method used for the culture of jejunum organoids from 21-day-old suckling and 35-day-old weaned piglets (*n* = 12 per group). **(B)**. Histological observations, villus height and crypt depth of jejunum sections (stained with Alcian blue and Periodic Acid Schiff) from suckling and weaned piglets (*n* = 12 per group). Scale bars represent 100 µm. **(C)**. Mean Log2 fold change of the relative abundance of metabolites in jejunum contents of weaned versus suckling piglets (*n* = 12 per group). Significant differences (*p* < 0.05) are indicated in blue (metabolites more abundant in weaned piglets) or in green (metabolites more abundant in suckling piglets). **(D)**. Observed OTUs and Shannon *a*-diversity index and Non-Metric Dimensional Scaling (nMDS) two-dimensional representation of the microbiota *ß*-diversity using Bray-Curtis distance calculation (stress = 0.11) in the jejunum contents from suckling and weaned piglets (*n* = 12 per group). **: *p* < 0.01, ***: *p* < 0.001.

Our second objective was to determine if jejunum organoids from suckling and weaned piglets retained a developmental stage-specific phenotype (Figure 4A). *In vivo*, we observed, as expected, differences in the jejunum morphology, luminal metabolome and microbiota between suckling and weaned piglets. Villus height decreased after weaning while crypt depth increased after weaning (Figure 4B). The luminal relative concentration of 7 amino acids and succinate was higher in the jejunum of suckling piglets while the relative concentration of glucose, maltose, three amino acids and choline was higher in weaned piglets (Figure 4C and Supplementary Table S6). The Shannon index of *a*-diversity was higher in the jejunum of suckling piglets while the richness was not different (Figure 4D). A distinct microbiota structure was observed between suckling and weaned piglets but these differences were mainly related to the higher abundance of a single family (Peptostreptococcaceae) in the jejunum of suckling piglets (7.0%) when compared to weaned piglets (2.2%) (Figure 4D and Supplementary Table S7).

**FIGURE 5:**
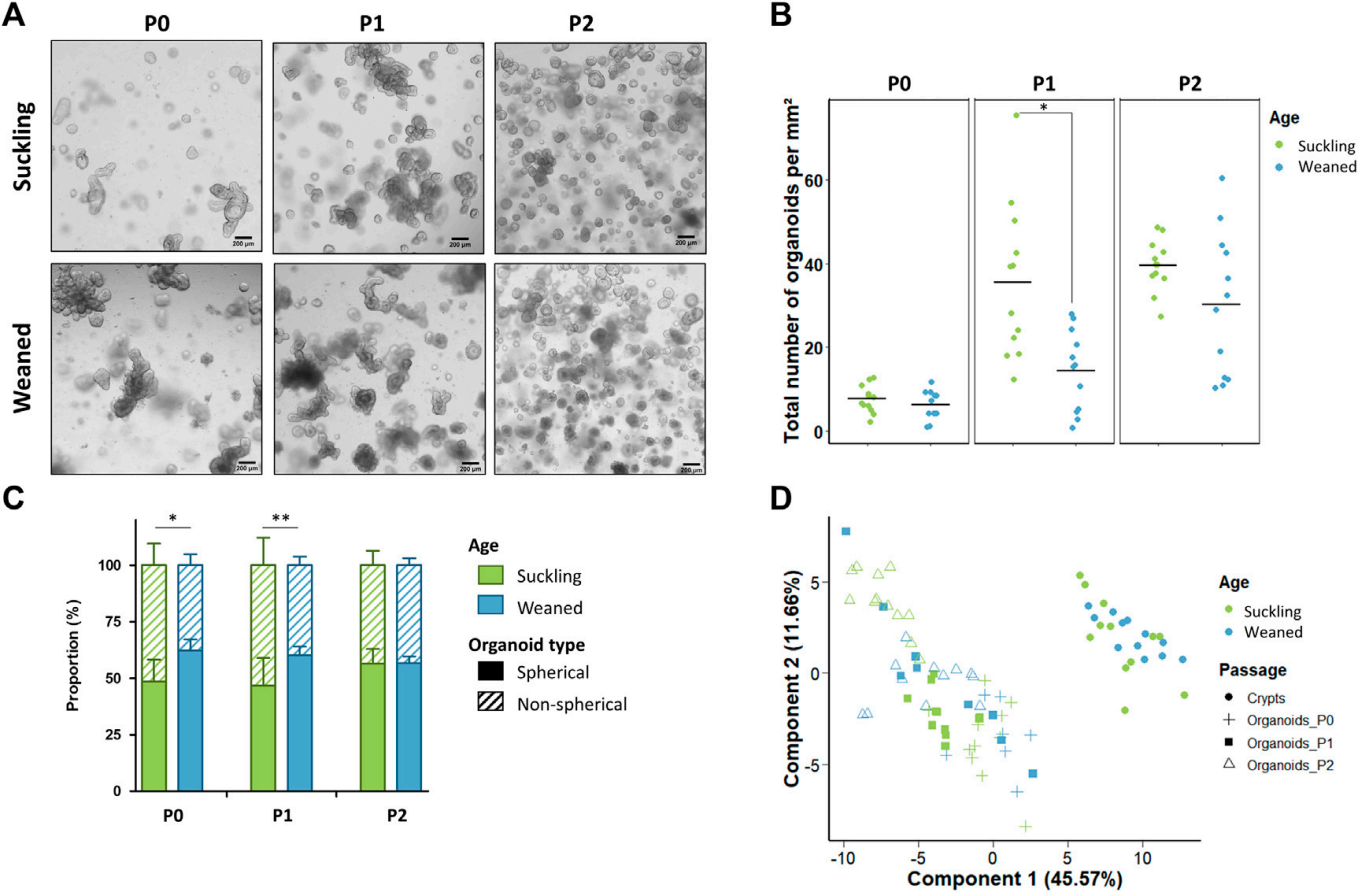
Characteristics of jejunum organoids from 21-day-old suckling and 35-day-old weaned piglets. **(A)**. Representative images showing jejunum organoids from 21-day-old suckling and 35-day-old weaned piglets at each passage. The scale bar represents 200 µm. **(B)**. Total number of jejunum organoids per mm^2^ at primary culture (*n* = 12 suckling, *n* = 11 weaned), passage 1 (*n* = 12 suckling, *n* = 12 weaned) and passage 2 (*n* = 12 suckling, *n* = 12 weaned). **(C)**. Proportion of organoids according to their spherical or non-spherical morphology at each passage. **(D)**. Principal component analysis (PCA) plot of the expression of 89 genes in jejunum crypts (*n* = 12 suckling, *n* = 11 weaned) and organoids at primary culture (*n* = 11 suckling, *n* = 7 weaned), passage 1 (*n* = 11 suckling, *n* = 9 weaned) and passage 2 (*n* = 12 suckling, *n* = 12 weaned). *: *p* < 0.05, **: *p* < 0.01.

Jejunum organoids were obtained from all suckling and weaned piglets (n = 12 per group) (Figure 5A). The density of organoids derived from the jejunum of suckling and weaned piglets was similar at P0 (Figures 5A,B). At P1, the density of organoids increased and was higher in organoids derived from suckling piglets while no difference was observed at P2. Spherical and non-spherical organoids were obtained from jejunum of suckling and weaned piglets at each passage. At primary culture P0 and passage P1, jejunum organoids were more spherical when derived from weaned piglets than from suckling piglets, while no difference was observed at P2 (Figure 5C).

**FIGURE 6:**
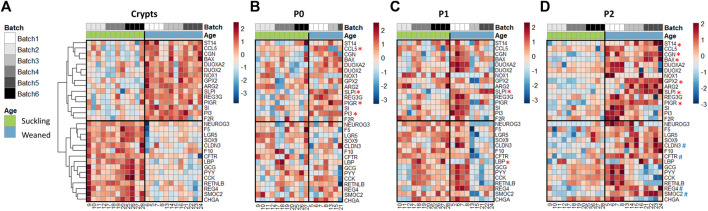
Gene expression profiles in jejunum crypts and organoids from 21-day-old suckling and 35-day-old weaned piglets. Heatmap representing the relative expression of genes (rows) in 21-day-old suckling and 35-day-old weaned piglet (columns) in jejunum crypts (*n* = 12 per group) **(A)**, organoids at primary culture (*n* = 11 suckling, n = 7 weaned) **(B)**, passage 1 (*n* = 11 suckling, *n* = 9 weaned) **(C)** and passage 2 (*n* = 12 suckling, *n* = 12 weaned) **(D)**. The numbers at the bottom are piglet identifiers. The colors represent the Z-scores (row-scaled relative expression) from low (blue) to high values (red). Genes (rows) were clustered in crypts and presented in the same order in organoids. *: significant difference in organoids in the same direction as observed in crypts. ^#^: significant difference in organoids in the opposite direction of the observed in crypts.

We analyzed the expression of 89 genes in jejunum crypts from suckling and weaned piglets and in the derived organoids at P0, P1 and P2. PCA revealed a distinct gene expression profile between crypts and organoids (axis 1, 46% of explained variance) and between suckling and weaned piglets but to a much lesser extent (axis 4, 6% of explained variance) (Figure 5D and Supplementary Figure S5). In jejunum crypts, 30 out of the 89 genes analyzed (34%) were differentially expressed between suckling and weaned piglets (Figure 6A and Supplementary Table S8). Genes more expressed in the jejunum crypts of suckling piglets (15 DEG) coded for markers of enteroendocrine cells (*NEUROG3, CHGA, CCK, PYY, GCG*), markers of stem cells and proliferation (*SMOC2, LGR5, SOX9, REG4*), a tight junction protein (*CLDN*3), a transporter of electrolytes (*CFTR*), proteins involved in innate immunity (*LBP, RETNLB*) and proteins involved in proteolytic activity (*F5, F10*). Genes more expressed in the jejunum crypts of weaned piglets (15 DEG) coded for digestive enzymes (*ARG2, SI*), an antimicrobial peptide (*REG3G*), an immunoglobulin transporter (*PIGR*), redox enzymes (*GPX2, DUOX2, DUOXA2, NOX1*), proteins involved in proteolytic activity (*PI3, F2R, ST14, SLPI*), a cell junction protein (*CGN*), a chemokine (*CCL5*) and an apoptosis marker (*BAX*).

Only one of the DEG identified *in vivo* (*SLPI*, coding for a protease inhibitor regulating the epithelial barrier) (Ozaka et al., 2021) was also differentially expressed at each passage between jejunum organoids from suckling and weaned piglets and in the same direction as observed *in vivo* (Figures 6B–D and Supplementary Table S8). Some DEG identified between jejunum crypts of suckling and weaned piglets (*PI3, ST14, PIGR, GPX2, CGN, BAX, LBP, CLDN3, SMOC2, REG4, CFTR*) were differentially expressed between jejunum organoids from suckling and weaned piglets at one (P0 or P2) but not at all passages and not always in the same direction. We also observed that promoting organoid differentiation did not induce the developmental stage-specific gene expression patterns observed *in vivo* in the jejunum of suckling and weaned piglets (Supplementary figure 3A, C and Figure 6A). Overall, our results obtained in the jejunum from suckling and weaned piglets indicated that the developmental stage-specific phenotype did not persist stably *in vitro* in organoids.

## Discussion

Gene expression in intestinal epithelial cells vary across spatial and temporal scales under the influence of signals derived from the host and from the luminal environment (Gehart and Clevers, 2019; Heppert et al., 2021). In organoids, host-derived factors are, at least in part, replicated by the extracellular matrix scaffold and by the culture medium that includes regulators of key pathways such as WNT and BMP (Sato et al., 2011). In contrast, luminal signals such as nutrients, the gut microbiota and its metabolites are lacking in standard conditions of organoid culture. In our study, we observed major differences in gut microbiota and metabolome both between the jejunum and the colon and to a lesser extent between suckling and weaned piglets, in line with previous studies (Frese et al., 2015; Holman et al., 2017; De Rodas et al., 2018; Beaumont et al., 2021a). These differences between the *in vivo* and the *in vitro* microenvironment of epithelial cells are probably the main drivers of the distinct gene expression in pig intestinal organoids when compared to the epithelial crypts, as described in previous reports in pig, mouse and human organoids (Kraiczy et al., 2019; Navis et al., 2019; van der Hee et al., 2020; Mohammadi et al., 2021).

We observed that, despite being cultured in the same conditions, pig organoids derived from the jejunum or from the colon retained a significant fraction (∼50%) of the location-specific expression patterns observed *in vivo*, which is in agreement with previous studies in human and mouse organoids (Middendorp et al., 2014; Kraiczy et al., 2019). In contrast, the developmental stage-specific gene expression profile observed in the crypts of the jejunum from suckling and weaned piglets was not retained in organoids. Differences in the mechanisms driving epithelial transcription according to location or developmental stage might explain why piglet organoids retained characteristics of the gut region but not of age. Indeed, the regional patterning of the intestinal epithelium begins *in utero*, independently of environmental cues (Middendorp et al., 2014; Thompson et al., 2018; Heppert et al., 2021). After birth, environmental plasticity of epithelial cells is mediated by epigenetic modifications induced notably by nutrients, the gut microbiota and its metabolites (Yu et al., 2015; Krautkramer et al., 2016, 2017; Pan et al., 2018; Heppert et al., 2021). Thus, environmental signals derived from the diet or from the gut microbiota might be required for the replication in pig intestinal organoids of a postnatal age-specific phenotype. The lower amplitude of the transcriptional differences observed during the suckling-to-weaning transition over 2 weeks when compared to gut regions might also explain why we did not observe the maintenance of a developmental-stage specific phenotype in piglet organoids.

Pattern recognition receptors (PRR) are key sensors of microbial ligands and their expression follows a specific pattern along the longitudinal axis of the intestine (Price et al., 2018; Arnaud et al., 2020; Kayisoglu et al., 2021). We observed that the location-specific expression profile of PRR (*TLR2, TLR4, NLRP6*) in piglet epithelial crypts was maintained in organoids, suggesting that their regional expression do not depend on microbial signals. Experiments in germ free mice and in embryo-derived organoids also indicated that the regional expression of most PRR do not depend on the gut microbiota (Price et al., 2018; Kayisoglu et al., 2021). Interestingly, the higher expression of *TLR5* in piglet crypts from jejunum than from colon was not stably maintained in piglet organoids, which is consistent with a previous study showing that epithelial expression of *TLR5* might be responsive to environmental cues (Price et al., 2018). PRR signaling in epithelial cells promotes the barrier function through the production of antimicrobial peptides and mucins, immunoregulatory responses, induction of enzymes producing reactive oxygen species and regulation of proteolytic activities (Peterson and Artis, 2014; Vergnolle, 2016; Campbell and Colgan, 2019). Although all of these components of epithelial innate immunity were previously shown to be highly responsive to the gut microbiota or their metabolic products (El Aidy et al., 2012; Larsson et al., 2012; Sommer and Bäckhed, 2015; Pan et al., 2018; Hansson, 2020), we observed that piglet organoids retained a regional patterning for antimicrobial peptides (*LYZ, REG3G, DEFB1, RETNLB*), a transmembrane mucin (*MUC1*), immunoregulatory proteins (*LBP, IL8, PIGR*), redox enzymes (*PTGS2, NOX1, GPX1*) and proteins involved in the regulation of proteolytic activity (*F10, MEP1B, PI3, SLPI*)*.* This microbiota-independent regional patterns are consistent with the minimal differences in gene expression observed between organoids derived from conventional and germ-free mice (Janeckova et al., 2019; Hausmann et al., 2020). Additional studies would be required to test whether the transcriptional differences between jejunum and colon organoids translate into functional differences. For instance, it would be interesting to test whether the higher expression of *TLR4* in pig colon organoids is associated with a higher responsiveness to lipopolysaccharides when compared to jejunum organoids, as described previously in human organoids (Kayisoglu et al., 2021).

Post-translational modification of histones is an important epigenetic mechanism driving regional gene expression in intestinal epithelial cells (Heppert et al., 2021). However, we observed that the segment-specific expression of *TLR4* and *LYZ* in the jejunum and colon organoids from suckling piglets was not associated with changes in promoter H3K4me3, which marks regions associated with higher transcriptional activation. The location-specific phenotype of pig intestinal organoids probably involves other epigenetic mechanisms, such as activity and/or level of segment-specific transcription factors or DNA-methylation, as demonstrated recently in human intestinal organoids (Kraiczy et al., 2019).

Regional patterning in the gut also supports the functional specialization of each segment for digestion, chemosensing and absorption of nutrients, water or electrolytes (Anderle et al., 2005; Gribble and Reimann, 2016; Lema et al., 2020). Piglet organoids retained the regional gene expression of some digestive enzymes (*SI*), nutrient transporters (*FAPB2, PEPT1*) and enterohormones (*CCK, GCG, CHGA*) which is consistent with previous studies in pig, human and mouse organoids (Middendorp et al., 2014; Zietek et al., 2015, 2020; Kozuka et al., 2017; Kraiczy et al., 2019; Barnett et al., 2021; Kayisoglu et al., 2021). Interestingly, some location-specific expression patterns (e.g. *LCT*, *GLUT2*) were observed only when piglet organoids were cultured in a medium lacking niche factors. This result is consistent with previous studies showing that differentiation of mouse or human organoids is required to reproduce the location-specific expression patterns of certain genes (Middendorp et al., 2014; Kraiczy et al., 2019; Zietek et al., 2020).

In mammals, the suckling-to-weaning transition is associated with a remodeling of the intestinal epithelial barrier function (Hooper, 2004; Pié et al., 2004; Ménard et al., 2008; Pan et al., 2018; Beaumont et al., 2020, 2022). In our study, jejunum organoids did not retain the weaning-induced upregulation of genes involved in epithelial barrier function (e.g. *DUOX2, NOX1, PIGR, REG3G, CCL5, SLPI*)*.* Major nutritional adaptations also occur in the small intestine epithelium at the suckling-to-weaning transition, as exemplified by the induction of sucrase-isomaltase (*SI*) and arginase-2 (*ARG2*) (Henning, 1985; Navis et al., 2019; Mohammad et al., 2020). We observed that jejunum organoids derived from suckling and weaned piglets did not retain the developmental stage-specific expression patterns of digestive enzymes (*SI, ARG2*), enteroendocrine cell markers (*CCK, PYY, GCG, CHGA, NEUROG3*) and an electrolyte transporter (*CFTR*), even when cultured in a medium promoting differentiation. These results are consistent with a previous study in piglets showing that organoids do not retain the enzymatic switch observed at weaning in the jejunum (Mohammad et al., 2020). Further studies will be needed to determine if pig organoids can retain developmental stage-specific characteristics in other digestive segments (e.g. ileum or colon) or with more contrasted age differences (e.g. earlier weaning).

In our study, we observed a large proportion of spherical organoids, which is consistent with a proliferative phenotype (Merker et al., 2016) also revealed by the high density of organoids after the first passage. Interestingly, we observed some region and age-related differences in organoid morphology and density, which could be linked to the differential expression observed *in vivo* of stem cell markers (*LGR5, SOX9, SMOC2, OLFM4*) between the jejunum and the colon and between suckling and weaned piglets. As discussed above, promoting differentiation might help to better reproduce the regional patterns of the gut in pig organoids but further optimization of the culture conditions might also help to improve the physiological relevance of the model. For instance, based on our metabolomics data, we propose that adding gut microbiota-derived metabolites such as short chain fatty acids to the culture medium of colon organoids could help to recreate *in vitro* the luminal environment of the pig large intestine, as performed before in human organoids (Freire et al., 2019; Pearce et al., 2020). Co-culture with region or developmental stage-specific bacteria, addition of nutrients derived from milk (e.g. lactose) or solid food might also help to reproduce more closely in organoids the phenotype observed *in vivo*.

In conclusion, we showed that organoids derived from the jejunum and colon of suckling and weaned piglets retained a location-specific phenotype but failed to reproduce the modifications of epithelial gene expression induced by the suckling-to-weaning transition over 2 weeks. Thus, piglet intestinal organoids represent a powerful model to study intestinal epithelium physiology in a gut region-specific manner. Our work also identified a set of genes which regional expression is maintained *in vitro* and that could be the focus of future studies with pig organoids. Further improvement of the culture conditions, notably by replicating more closely the luminal environment, might help to improve the physiological relevance of the intestinal organoid model.

## Data Availability

The datasets presented in this study can be found in online repositories. The names of the repository/repositories and accession number(s) can be found below: https://www.ncbi.nlm.nih.gov/, PRJNA800253.

## Abbreviations

DEG: Differentially Expressed Genes; D21: Postnatal day 21, D35: Postnatal day 35; DMEM: Dulbecco’s Modified Eagle Medium; FBS: fetal bovine serum; H3K4me3: Trimethylation of lysine four of Histone three; PRR: Pattern Recognition Receptor. TSS: transcription start site.
