# Supplementary material for: The phenotype of the gut region is more stably retained than developmental stage in piglet intestinal organoids
Source: Front Cell Dev Biol. 2022 Aug 29;10:983031. doi: 10.3389/fcell.2022.983031 (PMC9465596; doi:10.3389/fcell.2022.983031)
Supplement: Supplementary file 2 [file DataSheet1.DOCX]

Supplementary Material


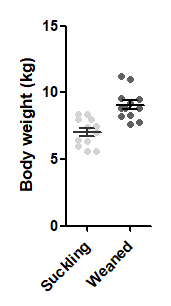


**Supplementary Figure 1:** Body weight of 21-day-old suckling piglets (n=12) and 35-day-old weaned piglets (n=12)

**
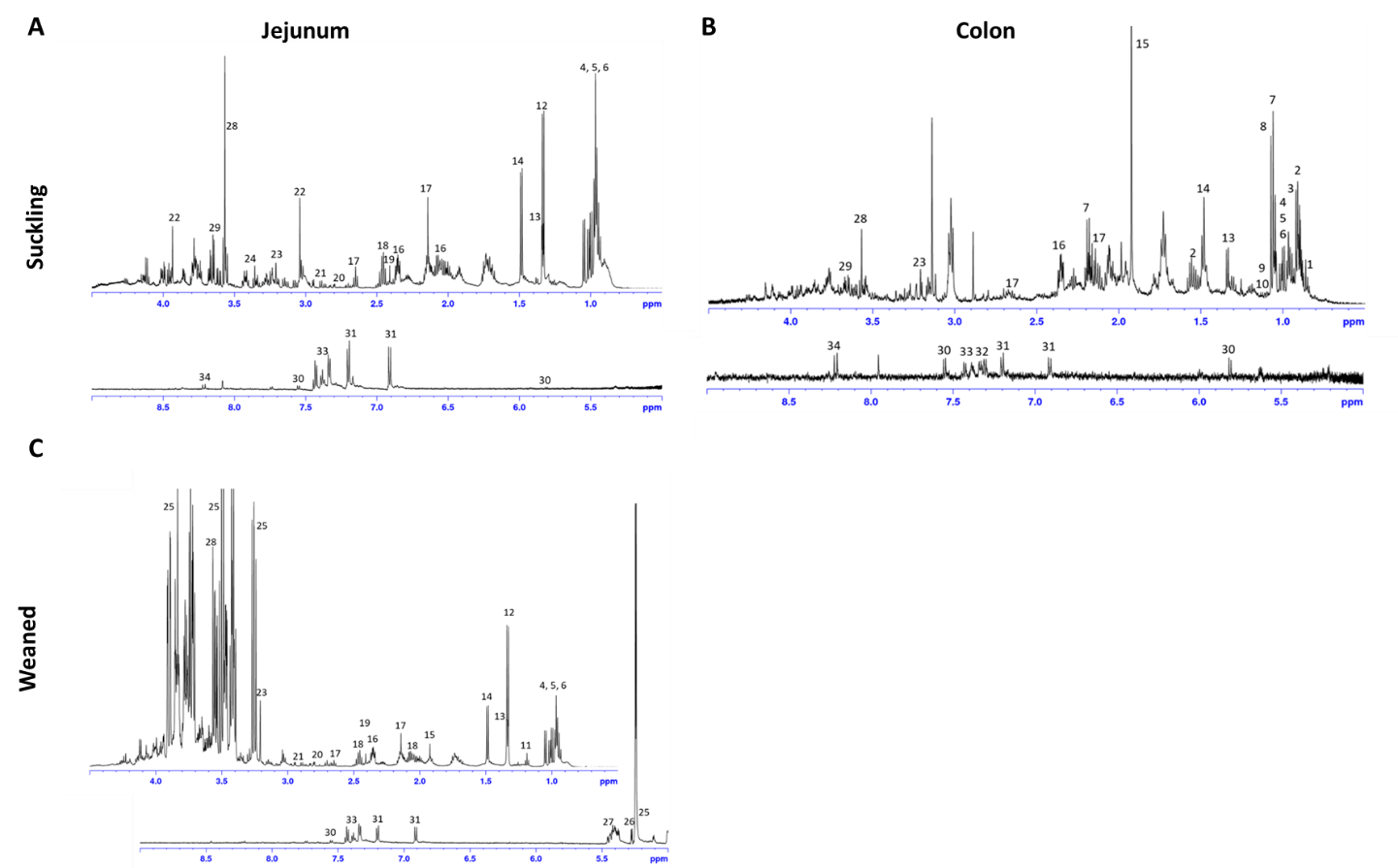
**

**Supplementary Figure 2:** Aliphatic region (δ^1^H 4.5 – 0.5 ppm) and aromatic region (δ^1^H 9 – 5 ppm) of a representative NMR spectrum of the jejunum content of 21-day-old suckling piglets (**A**), colon content of 21-day-old suckling piglets (**B**), jejunum content of 35-day-old weaned piglets (**C**). The numbers indicate identified metabolites described in supplementary table S2.

**
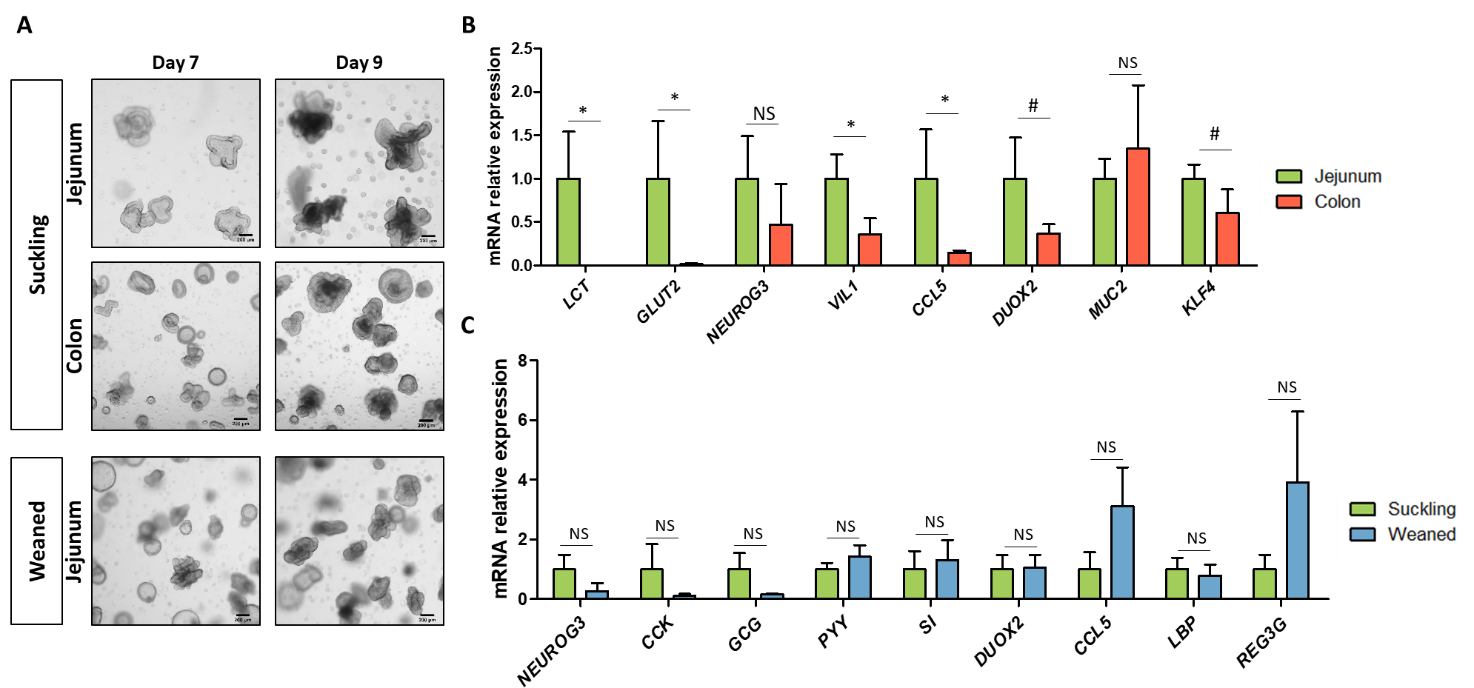
**

**Supplementary Figure 3: Effects of differentiation on gene expression in piglet organoids. A.** Representative images showing jejunum and colon organoids from 21-day-old suckling piglet and jejunum organoids from 35-day-old weaned piglets at day 7 (IntestiCult medium) and at day 9 (differentiation medium). The scale bar represents 200 µm. **B**. Gene expression in differentiated jejunum and colon organoids derived from 21-day-old suckling piglets (n=4 per group). **C**. Gene expression in differentiated jejunum organoids derived from 21-day-old sucking or 35-day-old weaned piglets (n=4 per group). *: significant difference in organoids in the same direction observed in crypts. #: significant difference in organoids in the opposite direction observed in crypts. NS: not significant.

**
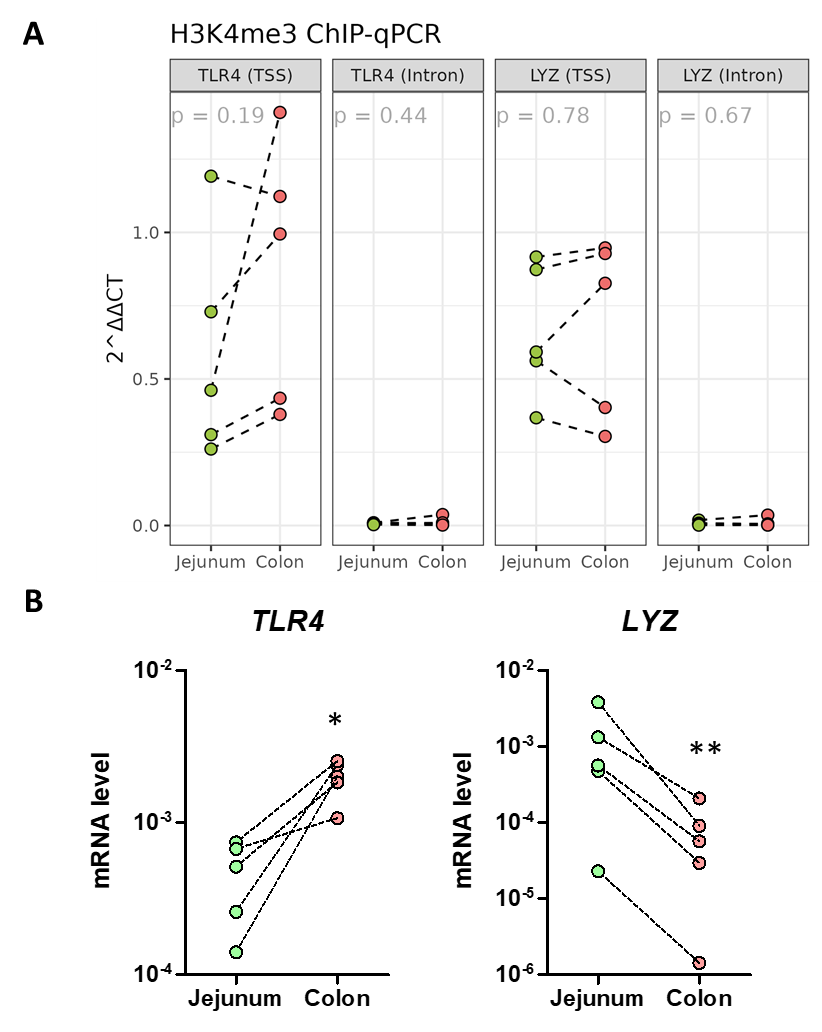
**

**Supplementary Figure 4: Epigenetic regulation of the regional expression of *TLR4* and *LYZ* in jejunum and colon organoids from suckling piglets. A.** ChIP-qPCR was used to quantify the trimethylation of histone H3 lysine 4 (H3K4me3) in jejunum and colon organoids from 21-day-old suckling piglet (n=5 per group). H3K4me3 was analyzed in the promoter region (TSS) and intronic region of *TLR4* and *LYZ*. H3K4me3 in the TSS of the housekeeping gene HPRT was used for normalization. Numbers on the figure are piglet identifiers. The dotted lines link jejunum and colon samples from the same piglet. **B**. Log-10 transformed expression of *TLR4* and *LYZ* in the organoids used for ChIP-qPCR experiments (n=5 per group). Groups were compared with paired t-tests. *: p<0.05, **: p<0.01.

**
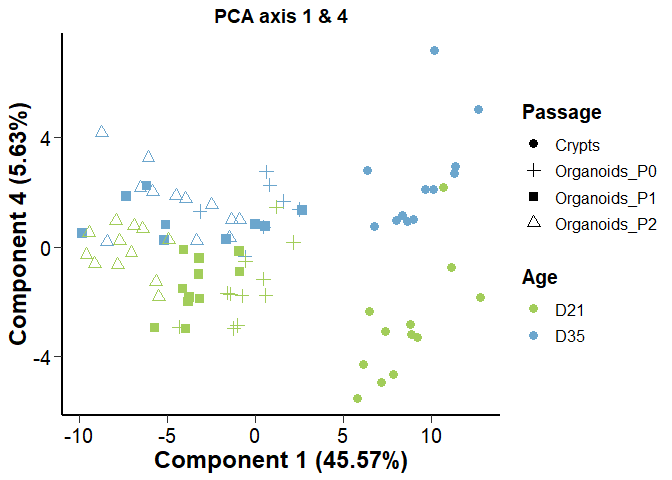
**

**Supplementary Figure 5: Gene expression in jejunum organoids from 21-day-old suckling and 35-day-old weaned piglets.** Principal component analysis (PCA) plot of the expression of 89 genes in jejunum crypts (n=12 suckling, n=11 weaned) and organoids at primary culture (n=11 suckling, n=7 weaned), passage 1 (n=11 suckling, n=9 weaned) and passage 2 (n=12 suckling, n=12 weaned). The axes 1 and 4 are presented.

**Table S1:** List of oligonucleotides used for gene expression analysis using qPCR

**Table S2:** Metabolites identified by NMR metabolomics in the jejunum (J) and colon (C) content of 21-day-old suckling and 35-day-old weaned piglets. “*” indicates the peak used for quantification based on the corresponding bucket intensity (not overlapping with peaks from other metabolites). Multiplicity of signals is indicated within brackets: s, singlet; d, doublet; t, triplet; m, multiplet.

**Table S3:** Relative concentration of metabolites in the jejunum and colon contents from 21-day-old suckling piglets

**Table S4:** Relative abundance of bacterial phyla and families in the jejunum and colon contents from 21-day-old suckling piglets

**Table S5:** Relative expression of mRNA in the jejunum and colon crypts and organoids from 21-day-old suckling piglets

**Table S6:** Relative concentration of metabolites in the jejunum contents from 21-day-old suckling and 35-day-old weaned piglets

**Table S7:** Relative abundance of bacterial phyla and families in the jejunum contents from 21-day-old suckling and 35-day-old weaned piglets

**Table S8:** Relative expression of mRNA in the jejunum crypts and organoids from 21-day-old suckling and 35-day-old weaned piglets
